# The prevalence of foot ulcers in diabetic patients in Pakistan: A systematic review and meta-analysis

**DOI:** 10.3389/fpubh.2022.1017201

**Published:** 2022-10-25

**Authors:** Sohail Akhtar, Aqsa Ali, Sadique Ahmad, Muhammad Imran Khan, Sajid Shah, Fazal Hassan

**Affiliations:** ^1^Department of Mathematics and Statistics, University of Haripur, Haripur, Pakistan; ^2^Department of Statistics, Government College University, Lahore, Pakistan; ^3^EIAS, Data Science and Blockchain Laboratory, College of Computer and Information Sciences, Prince Sultan University, Riyadh, Saudi Arabia

**Keywords:** pooled prevalence, foot ulcer, diabetes, Pakistan, meta-analysis, systematic review

## Abstract

We aimed to determine the pooled prevalence of diabetic foot ulcers in Pakistan. MEDLINE (PubMed), Web of Science, Google scholars, and local databases were systematically searched for studies published up to August 10, 2022, on the prevalence of foot *ulcers* among diabetic patients in Pakistan. Random-effects meta-analysis was used to generate summary estimates. Subgroup analysis and meta-regression models were used to address the issue of high heterogeneity. Two authors independently identified eligible articles, collected data, and performed a risk of bias analysis. Twelve studies were included in the meta-analysis (14201, range 230–2199, diabetic patients), of which 7 were of “high” quality. The pooled prevalence of diabetic foot ulcers was 12.16% (95% CI: 5.91–20.23%). We found significant between-study heterogeneity (I^2^ = 99.3%; *p* < 0.001) but no statistical evidence of publication bias (*p* = 0.8544). Subgroup meta-analysis found significant differences in foot ulcer prevalence by publication year and by the duration of diabetes. An increasing trend was observed during the last two decades, with the prevalence of diabetic foot ulcers being the highest in the latest period from 2011 to 2022 (19.54%) than in the early 2000 s (4.55%). This study suggests that the prevalence of diabetic foot ulcers in Pakistan is relatively high, with significant variation between provinces. Further study is required to identify ways for early detection, prevention, and treatment in the population.

## Introduction

A diabetic foot ulcer is a chronic consequence of diabetes characterized by lesions in the deep tissues. It causes neurological problems and peripheral vascular diseases in the lower extremities (1, 2). It poses a significant challenge for societies worldwide (3, 4). Foot ulceration and infection reduce patients' quality of life and significantly increase their risk of amputation, which is a tragic end for most people (4). It is an expensive disease to treat. Currently, 537 million adults are living with diabetes. This figure is forecast to increase to over 783 million adults by 2045 (5). Throughout their lives, 25% of adults will develop foot ulcers (6). Diabetes-related foot and lower limb issues are severe and long-lasting. They affect 40–60 million people with diabetes around the world. Chronic foot ulcers and amputations among diabetic patients significantly reduce the quality of life and increase mortality risk (6). Diabetes foot is one of the most common, costly, and severe diabetic complications. Amputation is 10–20 times more common in people with diabetes than in non-diabetics. It is argued that a lower limb or part of a lower limb is amputated globally every 30 s due to diabetes (6). Particularly in low-income regions, diabetic foot ulcers can have a significant economic, social, and public health impact without an appropriate educational program and adequate and appropriate footwear (6). The prevalence of foot ulcers among diabetic patients is 6.3% around the world. The highest prevalence is in Belgium at 16.6%, and in Asia, it is 5.5%. The lowest prevalence of foot ulcers in Australia is 1.5% (1).

The prevalence of diabetes and associated complications in Pakistan is steadily rising (7–9). According to the International Diabetes Foundation, 33 million (26.7%) people are living with diabetes (10). Diabetic foot ulcers and infections place a significant financial and resource strain on healthcare systems by requiring hospital in-patients and outpatients to be handled by primary care and community care services. In terms of overall performance, Pakistan is ranked 154th out of 195 countries (11). Pakistan, as a developing country, struggles to sustain an effective healthcare system in the form of quality healthcare, healthcare education, and accessibility (12). With the limited number of diabetic foot ulcer management centers, Pakistan is ill-equipped to address the problem of diabetes and diabetic foot ulcer complications. According to published studies, the prevalence of diabetic foot ulcers in Pakistan ranges from 2.1 (13) to 50.9% (14). The rising prevalence of diabetic foot ulcers in Pakistan prompted this study to identify systematically, select, characterize, summarize, and estimate the pooled prevalence of diabetic foot ulcers in Pakistan till August 10, 2022.

## Methods

### Search strategy

The PRISMA Guidelines (15) were followed in this study. Similarly, to our previous studies (16–18), two of us (S.A. and F.H.) identified articles on the prevalence of diabetic foot ulcers in Pakistan published from inception to August 10, 2022. We thoroughly searched electronic databases such as Medline (PubMed), Web of Science, Google Scholar, and local databases. The following keywords were combined to explore the potential articles: “diabetic feet” OR “DFUs” OR “diabetic foot” OR “diabetic foot ulceration” OR “diabetic foot problem” OR “diabetic foot ulcer” AND “epidemiology” OR “prevalence” AND “Pakistan” OR “Pakistani” as well as variations thereof. We also looked through the reference lists of the selected studies for other potentially relevant studies. The PRISMA Guidelines Checklist is attached in the Supplementary File S1.

### Inclusion and exclusion criteria

For this study, articles were included if they met the following criteria: (1) based on a population-based survey or hospital-based study published in English up to August 10, 2022; (2) participants must be Pakistan residents. The following articles were excluded if they were: (1) letters to the editor, reviews, case series, case studies, conference abstracts, qualitative studies, and intervention studies; (2) based on the Pakistani community living outside Pakistan; (3) did not report sufficient data; (4) were irrelevant to a diabetic foot ulcer, and (5) were based on duplicated information (data). Using a two-step procedure, the selection of articles was conducted. Two authors (S.A. and F.H.) separately examined the titles and abstracts of all identified articles. Second, the full texts of the pre-selected publications were independently evaluated based on the previously established inclusion criteria. When necessary, a third reviewer (A.A.) resolved conflicts.

### Data extraction

A prepiloted data collection form was used by two independent investigators (S.A. and A.A.) to collect data on the following variables: author first, publication year, survey year, study design, the geographical location where the study was performed, the average age of diabetic patients, total sample size, the proportion of men, the number of participants with foot ulcers, sampling strategy, and setting (rural vs. urban). Discrepancies and uncertainties were explored and resolved through cross-checking of the data.

### Study quality assessment

Two investigators (A.A. and F.H.) independently evaluated the risk of bias in the selected studies by adapting items from the JBI Critical Appraisal Checklist for Studies Reporting Prevalence Data (19). Disparities regarding methodological quality assessment scores were resolved by discussion and adjudication by a third author (SA). The studies were graded on a scale of 0 to 9. Using the score, we put each study into one of three categories: high risk (1–3), moderate risk (4–6), or low risk of bias (7–9).

### Statistical analysis

The statistical software R (version 4.2.1) was used to conduct all analyses, and a *P* value of 0.05 was considered statistically significant. For the statistical pooling of the prevalence of foot ulcers among diabetic patients, random effects (Der Simonian-Laird) models were used (20, 21). The Cochrane Q-statistic was utilized to test for statistical heterogeneity, and I^2^ was used to quantify it. Pooled results were presented with 95% confidence intervals (CIs) and a forest plot. Heterogeneity was defined as I^2^ >50% (22, 23). Publication bias was initially analyzed visually using a funnel plot and later statistically with the Egger regression and Beggs tests (24, 25). Subgroup analysis was conducted to find potential sources of heterogeneity in the case of large heterogeneity.

Subgroup meta-analyses were performed according to different extracted variables (participant age, gender, geographical region, and time period). To further explore heterogeneity, meta-regression analyses were performed to determine the association between the prevalence of foot ulcers and study characteristics. The covariates in the meta-regression considered were: year of publication, setting (urban vs rural), sample size, year of investigation, mean age of diabetic patients, methodological quality, and gender (male vs. female). To examine the impact of individual studies on the pooled prevalence estimates, sensitivity analyses were carried out by excluding each study. The agreement between the investigators was evaluated by the Kappa statistic (26).

## Result

**Figure 1:**
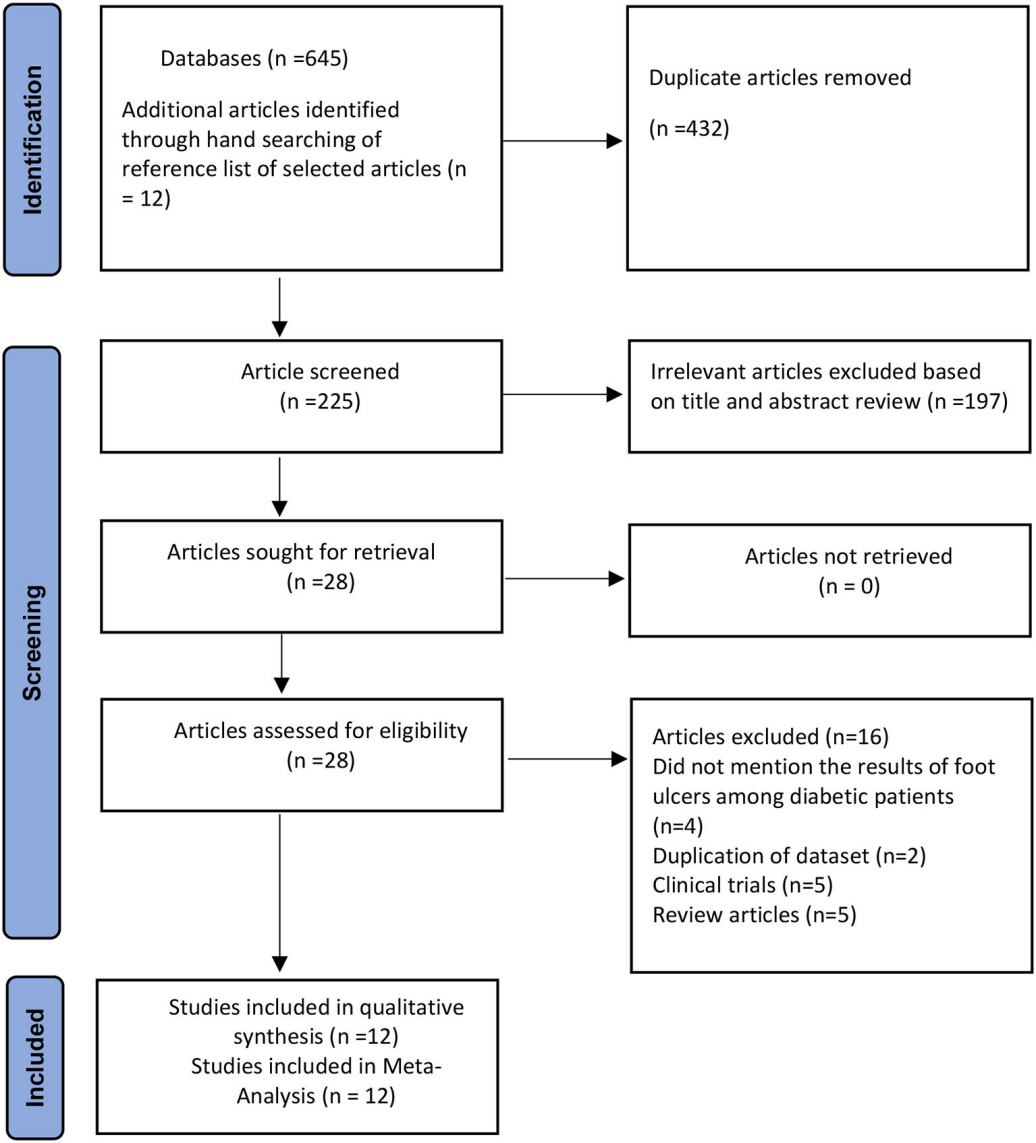
PRISMA flowchart of the prevalence of foot ulcer in diabetic patients in Pakistan (15).

Figure 1 displays the PRISMA selection and exclusion flowchart. A total of 657 studies were identified, including 645 via database searches and 12 from additional sources. After deduplication (*n* = 432), 197 studies were found ineligible after their titles and abstracts were thoroughly screened. The remaining 28 studies were subjected to a full-text evaluation to determine their eligibility; they were eliminated because they did not match the inclusion criteria. In the end, 12 papers were included in the analysis. The authors' inter-rater agreement for study inclusion was very good (Kappa = 0.83, *p* = 0.001).

Table 1 summarizes the key characteristics of the 12 studies included in this analysis. These articles included only Pakistani nationals, with sample sizes ranging from 230 (32) to 2199 (29), with a median of 1503 diabetic patients. Seven studies were conducted in Punjab province (13, 14, 30, 33–36), three studies were performed in Sindh (27–29), one was conducted in Azad Kashmir (31), and one study was conducted nationwide (32). Regarding the study design, a cross-sectional research design was utilized in 10 of the 12 studies; one study employed case-control, and the other used a prospective research design. Two studies were performed using convenient sampling procedures; one used simple random sampling techniques, one used cluster random cluster sampling; and the remaining four did not explicitly mention their sampling procedure. The reported foot ulcer prevalence rates in diabetic patients varied widely across provinces (Table 1). Ten studies were conducted on urban populations while two studies were conducted in both settings (urban and rural). The average participant age in the 11 studies providing this information was 52.29 years. The gender of the diabetic patients was provided in all papers. Regarding methodological quality bias, seven studies (27, 29–32, 35, 36) had a low risk of bias, five studies (13, 14, 28, 33, 34) had a moderate level, and none had a high risk of bias. The authors' agreement on the retrieved data was strong (Kappa score = 0.82, *p* = 0.001).

**Table 1:** General characteristics of studies selected in the systematic review (*n* = 12).

**Rerferences**	**Sample size**	**Cases**	**Prevalence (%)**	**Study design**	**Setting**	**Province**	**Sex**	**Working year**	**% Male**	**Average age**	**Sampling**	**Risk of bias**
Ahmed et al. (27)	500	20	4	Case-control	Urban	Sindh	Both	2004	32	55.2	Random Sampling	Low
Hashim et al. (13)	805	17	2.1	Cross-sectional	Urban	Punjab	Both	1999	47.2	49.26	NA	moderate
Khawaja et al. (28)	672	26	3.9	Cross-sectional	Urban	Sindh	Both	2000–2001	48.5	52.24	NA	moderate
Basit et al. (29)	2,199	229	10.4	Cross-sectional	Urban	Sindh	Both	1996–2001	48.5	51	**NA**	Low
Hussain et al. (30)	1,782	67	3.75	Case-control	Urban	Punjab	Both	2006–2008	73.13	55.7	NA	Low
Masood et al. (31)	318	22	6.9	Cross-sectional	Urban	Azad Kashmir	Both	2012	26.7	51.83	NA	Low
Khan et al. (32)	230	32	13.9	Cross-sectional	Urban	Pakistan	Both	2010–2011	40.86	53.82	Convenient	Low
Younis et al. (33)	1,940	136	7.02	Cross-sectional	Urban	Punjab	Both	2016–2017	37	51.24	NA	moderate
Khan et al. (34)	2,052	356	17.3	Cross-sectional	Urban	Punjab	Both	2017–2018	39.3	55	NA	moderate
Ejaz et al. (14)	1000	509	50.9	Cross-sectional	Urban	Punjab	Both	2017	66.6	48.26	Convenient	moderate
Akhtar et al. (35)	1,503	253	16.83	Cross-sectional	Both	Punjab	Both	2018–2019	33.53	51.58	Cluster Random	Low
Naseer et al. (36)	1,200	456	38	Cross-sectional	Both	Punjab	Both	NA	62.6	NA	NA	Low

## Quantitative synthesis

### Pooled prevalence of diabetic foot ulcers

**Figure 2:**
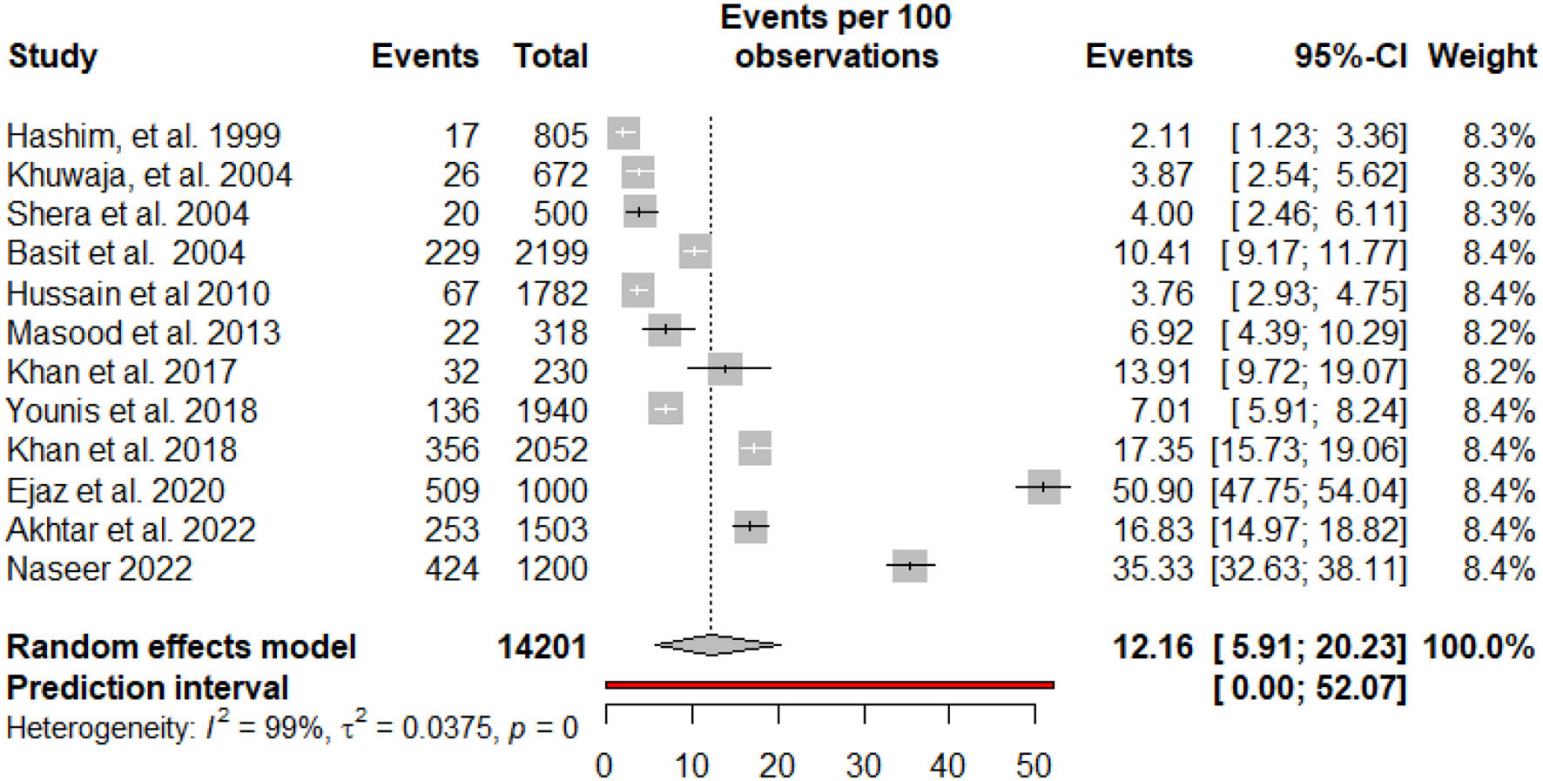
Forest plot of the prevalence of foot ulcers among diabetic patients in Pakistan.

**Figure 3:**
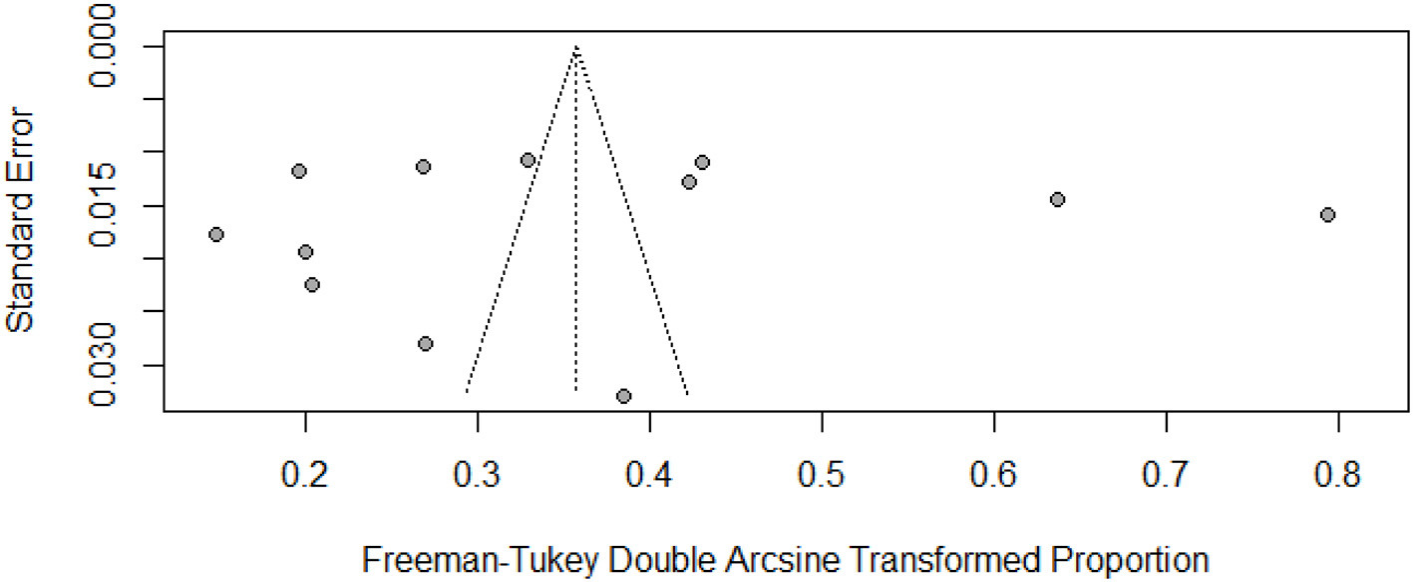
Funnel plot of the prevalence of foot ulcers among diabetic patients in Pakistan.

The pooled prevalence and subgroup meta-analysis for diabetic foot ulcers are summarized in Table 2. The prevalence of foot ulcers among diabetic patients was reported in 12 research articles (13, 14, 27–36) with a total of 14201 diabetic patients. The diabetic foot ulcer prevalence estimates in the included studies ranged from 2.11% (95% CI: 1.23–3.36%) to 50.90% (95% CI: 47.75–54.04%). The pooled prevalence of foot ulcers among diabetic patients was 12.16% (95% CI: 5.91–20.23%). The 95% prediction intervals were 0.0–52.07% (Figure 2). The I^2^ value (99.4%, *P* < 0.0001) indicated high between-study heterogeneity across the findings of different studies. The funnel plot (Figure 3), Begg's rank test (*z* = 0.41; *p* = 0.6808) and Egger's test (*t* = – 0.11; *p* = 0.9110) suggested no publication bias in the meta-analysis. The sensitivity analysis showed that the pooled prevalence of diabetic foot ulcers varied from 9.67% (95% CI: 5.23–15.28%) to 13.44% (95% CI: 6.68–22.06%) by excluding each study individually. The analysis found that no single study substantially affected the pooled prevalence of foot ulcers in diabetic patients.

**Table 2:** Summary estimates from meta-analyses of diabetic foot ulcers in Pakistan.

**Variable**	**No. of articles**	**No. of participants**	**No. of cases**	**Prevalence, (95% CI)**	**I^2^, %**	**95%, Prediction interval**	***P*** **-Value**			
							***Q* test**	**Egger test**	**Begg test**	**Subgroup difference**
Foot Ulcer in Diabetic patients	12	1,4201	2,123	12.16 (5.91–20.23)	99.4	0.00–53.07	< 0.001	0.911	0.6808	
**By Sex**								0.664	0.1857	0.3274
Male	6	3,755	459	12.04 (6.56–18.88)	96.2	0.00–41.71	< 0.001			
Female	5	4,680	484	7.29 (1.92–15.69)	98.7	0.00–51.67	< 0.001			
**Time period**								0.854	0.9379	0.0023
1999–2010	5	5,958	359	4.55 (2.35–7.42)	96.6	0.00–18.97	< 0.001			
2011–2022	7	7,043	1,308	19.54 (9.54–32.03)	99.3	0.00–69.76	< 0.001			
**Ulcer duration**										0.0491
≤ 10 years	3	4,440	291	6.16 (4.11–8.58)	87.6	0.00–54.37	< 0.001			
>10 years	2	1,202	307	26.60 (6.36–54.30)	99		< 0.001			
**By location**								0.205	0.7884	0.2335
Punjab	7	10,282	1,338	16.13 (5.57–30.79)	93.6	0.00–76.19	< 0.001			
Sindh	3	3,371	275	5.86 (2.51–10.48)	96	0.00–93.90	< 0.001			
Azad Kashmir	1	318	22	6.92 (4.36–9.99)						

To analyze the substantial sources of statistical heterogeneity, subgroup meta-analyses were conducted using age group, gender, geographical location, and time period. The subgroup meta-analysis based on geographical location showed that the prevalence of foot ulcers in diabetic patients was highest in studies conducted in Punjab province [16.13% (95% CI: 5.57–30.79%); *n* = 7], followed by Azad Kashmir and 6.92% (95% CI: 4.36–9.99; *n* = 1), and was lowest in Sindh (5.86% (95% CI: 2.51–10.48%; *n* = 3). When stratified by publication year, the pooled prevalence for diabetic foot ulcers estimates were 4.55% (95% CI: 2.37–7.42%; *n* = 5) from 1999 to 2010 and 19.54% (95% CI: 79.54–32.03%; *n* = 7) during 2011–2022. The highest prevalence of diabetic foot ulcers has been detected in recent years. When stratified by gender, the pooled prevalence of foot ulcers in male diabetic patients (12.04%; 95% CI: 3.48–18.88%; *n* = 6) was higher than in female diabetic patients (7.29%; 95% CI: 1.92–15.69%; *n* = 5).

The meta-regression analysis (Table 3) revealed that the prevalence of foot ulcers among diabetic patients significantly increased with the publication year (β = 0.0179; 95% CI: 0.0075–0.0282; *p* = 0.0007; R^2^ = 49.07%), as well as the year of investigation (β = 0.0144; 95% CI: 0.0021–0.0267; *p* = 0.0222; R^2^ = 29.93). The findings also showed that neither the percentage of men in the sample, the sample size, nor the methodological quality of the studies was significantly associated with the prevalence of foot ulcers in diabetic patients.

**Table 3:** Univariable meta-regression analyses.

**Variable**	**Beta (β)**	***p*-value**	**95% CI**	**R^2^%**
Publication Year	0.0179	0.0007	0.0075–0.0282	49.07
Year of investigation	0.0144	0.0222	0.0021–0.0267	29.93
Methodology	0.0155	0.8986	−0.2226– 0.2536	0.00
Male ratio	0.0052	0.1884	−0.0026–0.0130	6.17
Sample size	0.00	0.9152	−0.0003–0.0001	0.00

## Discussion

Over the last few decades, diabetes and its associated consequences have become more widespread. Diabetes-related hospitalizations are disproportionately impacted by foot ulcers, which account for half of the hospitalizations (37). The development of a diabetic foot ulcer is a significant predictive indication of mortality risk. Over half of patients who acquire a foot ulcer will die within 5 years, primarily from cardiovascular disease and diabetes complications (38). We did the first systematic review and meta-analysis to determine the pooled prevalence estimate of diabetic foot ulcers in Pakistan from January 1999 to August 2022. This study combined information from 12 distinct data sets involving 14201 diabetic patients from varied geographical regions of Pakistan. This study's findings will contribute to developing public health policies to reduce the prevalence of diabetic foot ulcers in Pakistan. The pooled rate of diabetic foot ulcers was 12.16% (95% CI: 5.91–20.23%). Wide variability is observed in the prevalence estimate across the studies, ranging from 2.1 to 50.9%. Significant heterogeneity is observed, which may be the reason for differences in sample size, year of study, and prevalence of diabetic neuropathy and peripheral artery disease.

Meta-analysis estimates were higher than those from Iran (39) and Saudi Arabia (3), where the prevalence rate of foot ulcers was 6.4 and 3.3%, respectively. This disparity could be attributed to a variation in research methodology. On the other hand, the prevalence of diabetic foot ulcers is lower than in the research conducted in Ethiopia at 13% (40), Sudan at 18.1% (41), and Spain at 17% (37). This disparity could be attributed to a variation in research methodology.

According to our data, male diabetic patients (12.04%) had more significant diabetic foot ulcers than female diabetic patients (7.29%). Males' harder physical labor could be one explanation for this gender discrepancy (42). The findings are congruent with those of a similar global survey (1). Our findings revealed that Punjab had the highest prevalence of diabetic foot ulceration (16.13%), while Sindh had the lowest (5.86%). All studies conducted in Sindh were published before 2004, which might be the reason for the lower prevalence in Sindh than Punjab. The results also revealed that the duration of a patient's diabetic disease is one of the risk factors for the development of foot ulcers. The probability of developing a foot ulcer increases as a patient's duration with diabetes increases. This is due to the medical condition's proclivity to worsen over time if not appropriately managed. This finding is similar to previous research, which indicated that diabetic foot ulcers worsened when individuals lived with diabetes for longer periods of time (39, 40).

The study has several benefits and drawbacks. We deployed exhaustive search procedures, rigorous selection criteria, and a dual review procedure. We could generate reliable prevalence estimates since the included studies provided sufficient data. Our analysis identified no evidence of publication bias, indicating that we did not overlook any papers that could have altered the results of our meta-analysis. Furthermore, due to their superior methodological quality, all included studies exhibited a low or moderate risk of bias. According to the meta-regression analysis, the methodological quality of the studies did not affect the assessment of the overall prevalence.

There are some limitations to this study. The meta-analysis revealed significant variation in the estimated pooled prevalence, as expected. To address the issue of substantial heterogeneity, subgroup analysis and meta-regression with components added to the univariate model were used. The outcomes of this study should be regarded with caution due to the significant degree of heterogeneity. Second, we could not discover any research article published on Khyber Pakhtunkhwa or Baluchistan. As a result, the findings should be regarded with caution. Thirdly, the aim of the study was to estimate the foot ulcers prevalence in diabetic patients which is the reason it excluded the studies which did not provide prevalence estimates. Fourthly, in the subgroup meta-analyses and meta-regression models, the choice of important covariates (HbA1c, peripheral artery diseases, smoking, and diabetic neuropathy) was limited, on the basis of the restricted availability of primary data in the eligible studies. Finally, because the number of papers included in this review is limited, a univariate meta-regression analysis rather than a multivariable meta-regression model is employed to assess the importance of each covariate.

## Conclusions

This study concludes with pooled estimates of foot ulcers among diabetic patients in Pakistan, indicating that diabetic foot is a substantial public health issue in Pakistan. The frequency of foot ulcers in the general population has increased over the past three decades, and this trend may continue in the future. Foot ulcer among diabetic patients is on the rise in Pakistan. Therefore, diabetic foot clinical centers are required for foot ulcer screening, identification, and management in urban as well as rural areas.

## Data availability statement

The original contributions presented in the study are included in the article/Supplementary material, further inquiries can be directed to the corresponding authors.

## Author contributions

All authors listed have made a substantial, direct, and intellectual contribution to the work and approved it for publication.

## Conflict of interest

The authors declare that the research was conducted in the absence of any commercial or financial relationships that could be construed as a potential conflict of interest.

## Publisher's note

All claims expressed in this article are solely those of the authors and do not necessarily represent those of their affiliated organizations, or those of the publisher, the editors and the reviewers. Any product that may be evaluated in this article, or claim that may be made by its manufacturer, is not guaranteed or endorsed by the publisher.

## References

[1] Zhang P Lu J Jing Y Tang S Zhu D Bi Y. Global epidemiology of diabetic foot ulceration: a systematic review and meta-analysis. Ann Med. (2017) 49:106–16. 10.1080/07853890.2016.1231932 27585063

[2] Katsilambros N. Who is the patient at risk for foot ulceration. In: N. Katsilambros, E. Dounis, P. Tsapogas, and N. Tentolouris, eds. Atlas of the Diabetic Foot. Chichester; Hoboken, NJ: Wiley (2003). p. 1–21 10.1002/047086138X

[3] Al-Rubeaan K Al Derwish M Ouizi S Youssef AM Subhani SN Ibrahim HM . Diabetic foot complications and their risk factors from a large retrospective cohort study. PLoS ONE. (2015) 10:e0124446. 10.1371/journal.pone.0124446 25946144 PMC4422657

[4] Jupiter DC Thorud JC Buckley CJ Shibuya N. The impact of foot ulceration and amputation on mortality in diabetic patients. I: from ulceration to death, a systematic review. Int Wound J. (2016) 13:892–903. 10.1111/iwj.12404 25601358 PMC7950078

[5] IDF Diabetes Atlas 10th Edition and other resources.

[6] https://idf.org/our-activities/care-prevention/diabetic-foot.html.

[7] Akhtar S Khan Z Rafiq M Khan A. Prevalence of type II diabetes in district dir lower in pakistan. Pakistan J Med Sci. (2016) 32:622. 10.12669/pjms.323.9795 27375702 PMC4928411

[8] Akhtar S Shah SW Javed S Alina A. Prevalence of diabetes and prediabetes in district swat Pakistan. J Pak Med Assoc. (2021) 71:243–6. 10.47391/JPMA.548 35157657

[9] Sun H Saeedi P Karuranga S Pinkepank M Ogurtsova K Duncan BB . Diabetes atlas: global, regional and country-level diabetes prevalence estimates for 2021 and projections for 2045. Diabetes Res Clin Pract. (2022) 183:109119. 10.1016/j.diabres.2021.109119 34879977 PMC11057359

[10] Akhtar S Nasir JA Abbas T Sarwar A. Diabetes in Pakistan: a systematic review and meta-analysis. Pakistan J Med Sci. (2019) 35:1173. 10.12669/pjms.35.4.194 31372163 PMC6659044

[11] https://borgenproject.org/facts-about-healthcare-in-pakistan/.

[12] https://hospaccxconsulting.com/healthcare-investment-opportunities-inpakistan-and-way-ahead/.

[13] Hashim R Khan FA Khan DA Shaukat A. Prevalence of macrovascular complications in diabetics of WAH, District Rawalpindi. J Pakistan Med Associat. (1999) 49:8–11. 10463008

[14] Ejaz F Ahmad A Hanif K. Prevalence of diabetic foot ulcer in lahore, Pakistan: a cross sectional study. Asian J Allied Health Sci. (2020):34–38. 10.52229/ajahs.v3i4.353

[15] Page MJ McKenzie JE Bossuyt PM Boutron I Hoffmann TC Mulrow CD . The PRISMA 2020 statement: an updated guideline for reporting systematic reviews. Syst Rev. (2021) 10:1–1. 10.1186/s13643-021-01626-4 33781348 PMC8008539

[16] Akhtar S Nasir JA Sarwar A Nasr N Javed A Majeed R . Prevalence of diabetes and pre-diabetes in Bangladesh: a systematic review and meta-analysis. BMJ Open. (2020) 10:e036086. 10.1136/bmjopen-2019-036086 32907898 PMC7482481

[17] Akhtar S Nasir JA Javed A Saleem M Sajjad S Khan M. The prevalence of type 2 diabetes in Afghanistan: a systematic review and meta-analysis. 10.1186/s12889-021-10993-5 PMC8130421 34001088

[18] Akhtar S Nasir JA Ali A Asghar M Majeed R Sarwar A. Prevalence of type-2 diabetes and prediabetes in Malaysia: a systematic review and meta-analysis. PLoS ONE. (2022) 17:e0263139. 10.1371/journal.pone.0263139 35085366 PMC8794132

[19] Munn Z Moola S Lisy K Riitano D Tufanaru C. Methodological guidance for systematic reviews of observational epidemiological studies reporting prevalence and cumulative incidence data. Int J Evid Based Healthc. (2015) 13:147–53. 10.1097/XEB.0000000000000054 26317388

[20] Higgins JP Thompson SG. Quantifying heterogeneity in a meta-analysis. Stat Med. (2002) 21:1539–58. 10.1002/sim.1186 12111919

[21] Barendregt JJ Doi SA Lee YY Norman RE Vos T. Meta-analysis of prevalence. J Epidemiol Commun Health. (2013) 67:974–8. 10.1136/jech-2013-203104 23963506

[22] Higgins JP Thompson SG Deeks JJ Altman DG. Measuring inconsistency in meta-analyses. BMJ. (2003) 327:557–60. 10.1136/bmj.327.7414.557 12958120 PMC192859

[23] Higgins JP Thomas J Chandler J Cumpston M Li T Page MJ . Cochrane Handbook for Systematic Reviews of Interventions. Hoboken: John Wiley & Sons (2019). 10.1002/9781119536604 PMC10284251 31643080

[24] Egger M Smith GD Schneider M Minder C. Bias in meta-analysis detected by a simple, graphical test. BMJ. (1997) 315:629–34. 10.1136/bmj.315.7109.629 9310563 PMC2127453

[25] Begg CB Mazumdar M. Operating characteristics of a rank correlation test for publication bias. Biometrics. (1994) 50:1088–101. 10.2307/2533446 7786990

[26] Viera AJ Garrett JM. Understanding interobserver agreement: the kappa statistic. Fam Med. (2005) 37:360–3. 15883903

[27] Ahmed U. Prevalence of chronic complications and associated factors in type 2 diabetes. J Pak Med Assoc. (2004) 54:54–9. 15134204

[28] Khuwaja AK Rafique G White F Azam SI. Macrovascular complications and their associated factors among persons with type 2 diabetes in Karachi, Pakistan—a multi-center study. J Pak Med Assoc. (2004) 54:60. 15134205

[29] Basit A Hydrie MZ Hakeem R Ahmedani MY Masood Q. Frequency of chronic complications of type 2 diabetes. J Coll Physicians Surg Pak. (2004) 14:79–83. 15228868

[30] Hussain F Sheikh MA Jamil A Nawaz H. Advanced glycation end-products and foot ulceration in type 2 diabetic patients: a case control study. Int J Agric Biol. (2010) 12:91–5.

[31] Masood CT Afzal W. Long-term complications of diabetes and co-morbidities contributing to atherosclerosis in diabetic population of Mirpur, Azad Kashmir. J Pak Med Assoc. (2013) 63:1383–6. 24392524

[32] Khan A Junaid N. Prevalence of diabetic foot syndrome amongst population with type 2 diabetes in Pakistan in primary care settings. JPMA. (2017) 67:1818–24. 29256523

[33] Younis BB Shahid A Arshad R Khurshid S Ahmad M Yousaf H. Frequency of foot ulcers in people with type 2 diabetes, presenting to specialist diabetes clinic at a Tertiary Care Hospital, Lahore, Pakistan. BMC Endocr Disord. (2018) 18:1–6. 10.1186/s12902-018-0282-y 30081878 PMC6090692

[34] Khan MI Azhar U Zubair F Khan ZA. Can we link foot ulcer with risk factors in diabetics? A study in a tertiary care hospital. Pakistan J Med Sci. (2018) 34:1375. 10.12669/pjms.346.16199 30559788 PMC6290204

[35] Akhtar S Ahmed OS Sarwar A Alina A Khan MI. Prevalence of foot ulcers in diabetic patients in Punjab, Pakistan. Front. Public Health. (2022) 10:967733. 10.3389/fpubh.2022.967733 36016895 PMC9397578

[36] Naseer S Malkera A Khan N Siddiqui AH Khan SA Ali S . Prevalence of diabetic complications in urban and rural population of Punjab. Pakistan J Med Health Sci. (2022) 16:69. 10.53350/pjmhs2216369

[37] Dòria M Rosado V Pacheco LR Hernández M Betriu À Valls J . Prevalence of diabetic foot disease in patients with diabetes mellitus under renal replacement therapy in Lleida, Spain. BioMed Res Int. (2016) 2016:7217586. 10.1155/2016/7217586 27190996 PMC4848423

[38] Jeyaraman K Berhane T Hamilton M Chandra AP Falhammar H. Mortality in patients with diabetic foot ulcer: a retrospective study of 513 cases from a single Centre in the Northern Territory of Australia. BMC Endocrine Disorders. (2019) 19:1–7. 10.1186/s12902-018-0327-2 30606164 PMC6318899

[39] Yazdanpanah L Shahbazian H Nazari I Arti HR Ahmadi F Mohammadianinejad SE . Prevalence and related risk factors of diabetic foot ulcer in Ahvaz, south west of Iran. Diabetes Metabolic Syndrome Clin Res Rev. (2018) 12:519–24. 10.1016/j.dsx.2018.03.018 29602761

[40] Tolossa T Mengist B Mulisa D Fetensa G Turi E Abajobir A. Prevalence and associated factors of foot ulcer among diabetic patients in Ethiopia: a systematic review and meta-analysis. BMC Public Health. (2020) 20:1–4. 10.1186/s12889-019-8133-y 31924173 PMC6954527

[41] Almobarak AO Awadalla H Osman M Ahmed MH. Prevalence of diabetic foot ulceration and associated risk factors: an old and still major public health problem in Khartoum, Sudan? Ann Translat Med. (2017) 5:340. 10.21037/atm.2017.07.01 28936434 PMC5599292

[42] Moura Neto A Zantut-Wittmann DE Fernandes TD Nery M Parisi MC. Risk factors for ulceration and amputation in diabetic foot: study in a cohort of 496 patients. Endocrine. (2013) 44:119–24. 10.1007/s12020-012-9829-2 23124278

